# Intrinsically Conductive Polymers for Striated Cardiac Muscle Repair

**DOI:** 10.3390/ijms22168550

**Published:** 2021-08-09

**Authors:** Arsalan Ul Haq, Felicia Carotenuto, Fabio De Matteis, Paolo Prosposito, Roberto Francini, Laura Teodori, Alessandra Pasquo, Paolo Di Nardo

**Affiliations:** 1 Dipartimento di Scienze Cliniche e Medicina Traslazionale, Università degli Studi di Roma “Tor Vergata”, Via Montpellier 1, 00133 Rome, Italy; arsalan.ulhaq@students.uniroma2.eu; 2 CIMER—Centro di Ricerca Interdipartimentale di Medicina Rigenerativa, Università degli Studi di Roma “Tor Vergata”, Via Montpellier 1, 00133 Rome, Italy; fabio.dematteis@uniroma2.it (F.D.M.); paolo.prosposito@roma2.infn.it (P.P.); roberto.francini@roma2.infn.it (R.F.); teodori@med.uniroma2.it (L.T.); 3 Department of Fusion and Technologies for Nuclear Safety and Security, Diagnostic and Metrology (FSN-TECFIS-DIM), ENEA, CR Frascati, 00044 Rome, Italy; alessandra.pasquo@enea.it; 4 Dipartimento di Ingegneria Industriale, Università degli Studi di Roma “Tor Vergata”, Via del Politecnico, 00133 Roma, Italy; 5 L.L. Levshin Institute of Cluster Oncology, I.M. Sechenov First Moscow State Medical University, 119991 Moscow, Russia

**Keywords:** conductive polymers, striated muscle cell electrical coupling, cardiac tissue engineering, cardiac muscle repair, electrical signals, biomimetic material constructs

## Abstract

One of the most important features of striated cardiac muscle is the excitability that turns on the excitation-contraction coupling cycle, resulting in the heart blood pumping function. The function of the heart pump may be impaired by events such as myocardial infarction, the consequence of coronary artery thrombosis due to blood clots or plaques. This results in the death of billions of cardiomyocytes, the formation of scar tissue, and consequently impaired contractility. A whole heart transplant remains the gold standard so far and the current pharmacological approaches tend to stop further myocardium deterioration, but this is not a long-term solution. Electrically conductive, scaffold-based cardiac tissue engineering provides a promising solution to repair the injured myocardium. The non-conductive component of the scaffold provides a biocompatible microenvironment to the cultured cells while the conductive component improves intercellular coupling as well as electrical signal propagation through the scar tissue when implanted at the infarcted site. The in vivo electrical coupling of the cells leads to a better regeneration of the infarcted myocardium, reducing arrhythmias, QRS/QT intervals, and scar size and promoting cardiac cell maturation. This review presents the emerging applications of intrinsically conductive polymers in cardiac tissue engineering to repair post-ischemic myocardial insult.

## 1. Introduction

Cardiovascular diseases (CVDs) represent the leading cause of death within the European Society of Cardiology member countries with 108.7 million active cases as of 2017 and 1.67 million lives lost to ischemic heart disease the same year [1]. On average, CVDs claim 17.3 million lives per year around the globe and this number is feared to soar to 23.6 million per year by 2030 [2]. CVDs also represent a massive economic burden for governments and families: about 1.48 trillion euros were spent in 2017 alone to cope with cardiovascular diseases, which was 9.81% of the total EU-28 GDP [3]. Myocardial infarction (MI) is one of the clusters of numerous other cardiovascular diseases and occurs due to the blockage of the coronary artery delivering blood (ischemia) to the ventricle with consequent oxygen shortage to the contractile cells (cardiomyocytes) [4]. The final result is that, after MI, billions of cardiomyocytes (CMs) with limited proliferation capacity are lost and substituted by heterogeneous collagen-rich fibrotic scar tissue [5,6]. Among others, the fibrotic scar does not display contractile capabilities and does not appropriately conduct electric currents, thus generating myocardial arrhythmias and asynchronous beating, contributing to determine heart failure in the worst-case scenario [5]. The current pharmacological approaches are palliative [7] and finalized to prevent intra-coronary blood clotting (thrombolytics, antiplatelet agents, such as aspirin, etc.) and post-ischemic ventricular dilation (ACE-inhibitors, β-blockers, etc.), but do not induce regeneration of the injured cardiac tissue. So far, the heart transplant is the gold standard in post-MI end-stage heart failure, but the lack of organ donors and the possibility of immune rejection makes this approach elusive [8].

**Figure 1:**
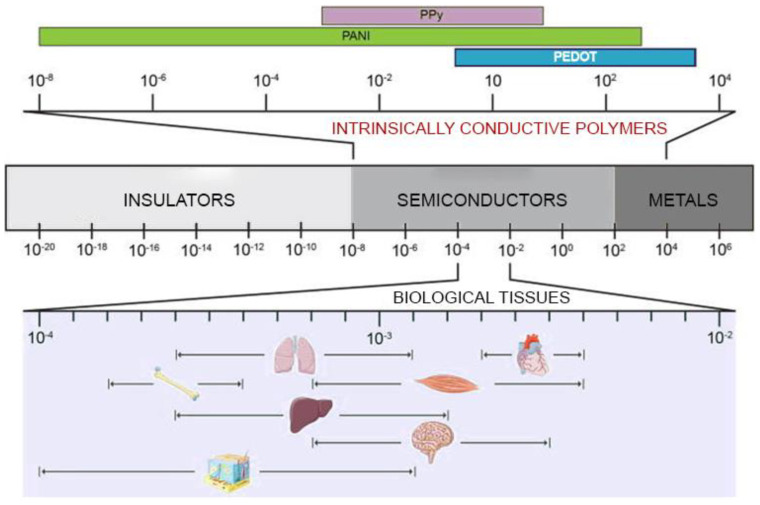
Electrical conductivity of common materials and biological tissues. The typical electrical conductivity values of intrinsically conductive polymers (top) are compared to those of common materials (centre) and biological tissues (bottom). Values are expressed in S/cm. Adapted with permission from [16].

**Figure 2:**
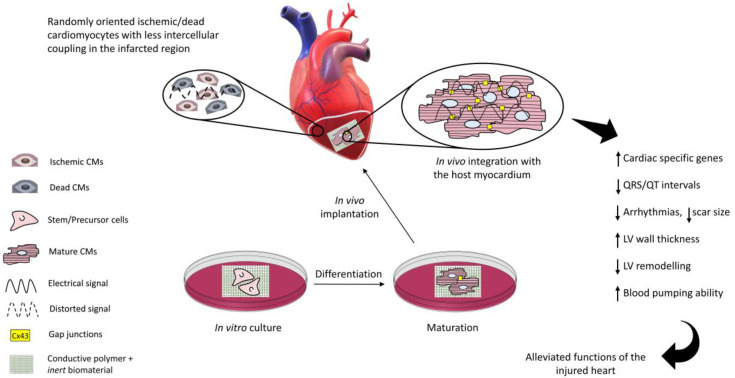
Conductive scaffold-based cardiac tissue engineering. After MI, damaged and dead cardiomyocytes (CMs) compromise the electrical conduction of the myocardium. The in vitro cell-laden construct can be transplanted at the infarction site to restore the electrical functions and to repair the damaged myocardium.

In recent years, the parallel progresses in cell biology, materials science, and advanced nano-manufacturing procedures have allowed us to envision the possibility to set up novel strategies (collectively dubbed “tissue engineering”) to combine cells and biomaterials to fabricate myocardium-like structures in vitro to be engrafted into the heart to repair the damaged parts. This approach is characterized by an extreme level of complexity and, as a matter of fact, after more than two decades of extensive efforts, many issues remain to be answered before tissue engineering products can be used at the bedside. Indeed, the maturation of CMs from stem cells is orchestrated by biological and physical signals released through the extracellular matrix (ECM) to the cells. ECM is a composite material made of a gel matrix and different fiber types. The interplay among the different ECM components releases physical signals relevant to determine the cell fate. Several studies have been performed to define protocols helpful to manufacture a structure (scaffold) emulating the native ECM on which cells can grow and differentiate. Natural or synthetic biomaterials, such as cardiac patches [9], injectable hydrogels [10], nanofiber composites [11], nanoparticles [12], and 3D hydrogel constructs [13], have been scrutinized to produce structures that mimic the mechanical properties of the extracellular matrix of the myocardium and potentially restoring the cardiac functions [14,15]. However, the issues related to arrhythmias and asynchronous beating of the injured myocardium are not resolved due to the non-conductive nature of most of the polymers used so far. The solution to this issue has been envisioned in conveying electric signals through scaffolds with embedded polymers displaying electroconductive characteristics comparable to the biological tissues (Figure 1) and this has already been demonstrated to enhance cell differentiation to mature CMs [16]. Electrically inert biomaterials can be blended with conductive polymers, such as polyaniline (PANI), polypyrrole (PPy), and Poly(3,4-ethylenedioxythiphene)/PEDOT, which belong to the family of intrinsically conductive organic materials [17]. The resulting electroconductive scaffold would harness the biocompatibility and the mechanical properties of the inert biomaterial and the electrical nature of the conductive component to drive the differentiation of the cultured stem/progenitor cells to cardiomyocyte-like cells with synchronous beating patterns, and enhance the expression of cardiac-specific genes, such as GATA4, Nkx2.5, cardiac troponins, and connexin 43 (C×43) [18,19,20]. GATA4 and Nkx2.5 are cardiac-specific transcription factors that play a fundamental role in myocardial differentiation and cardiac hypertrophy in the early stages of cardiogenesis in a developing embryo, while cardiac troponins are expressed in the cardiac muscles of the mammalian and avian species [21,22,23]. Connexin 43 is the principal gap junction protein of the heart which mediates action potential propagation between cells to synchronise cardiac contraction. In addition to this canonical role, it may act as a transcription regulator [24]. This cell-laden conductive scaffold improves the electrical signal propagation through the scar tissue, when implanted in vivo, and results in repair/regeneration of the injured myocardium with elevated ventricular wall thickness, improved blood pumping ability, reduced scar size, shorter QRS/QT intervals, and lower risk of arrhythmias, as shown in Figure 2 [25].

The present review is aimed at analyzing the state-of-the-art use of intrinsically electroconductive polymeric materials in the fabrication of functional cardiac constructs to envision future directions and potential clinical applications.

## 2. Conductive Polymer-Based Scaffolds in Cardiac Tissue Engineering

### 2.1. Polyaniline

Polyaniline (PANI) is a widely used material in tissue engineering and biomedical applications (Table 1) due to its intrinsic ability to conduct electric currents and good biocompatibility [26,27,28,29]. The polymer’s conductivity can be tuned by chemical or electrochemical doping (p-doping (oxidation) or n-doping (reduction)) [30]. However, the powdered form of the PANI does not dissolve in its doped form in any common organic solvents and the electro-mechanical properties of the blends and composites depend on the uniform dispersion of the PANI particle in the polymer matrix. PANI particles can be prepared previously and then added to the matrix. Alternatively, aniline is polymerised to polyaniline in the polymer matrix (in situ chemical polymerisation method) [31]. Different PANI blends and composites have been created using various synthesis methods and combining PANI with diverse biomaterials to manufacture suitable conductive constructs for cardiac repair.

The presence of PANI in these constructs improves the intercellular signaling pathways between cultured cells, which enhances the cell viability and various cellular behaviours as well. Good viability and proliferation rate were observed when H9c2 rat cardiac myoblasts were cultured on pristine and gelatin-blended polyaniline substrates. The cell population doubled after 54 h of culture [45] with smooth muscle-like morphology rich in microfilaments after six days [38]. Similarly, C2C12 myoblasts and bone marrow-derived mesenchymal stem cells proliferated well when cultured on polyaniline blended polyglycerol sebacate [42] and gelatin scaffolds [39]. The conductive scaffolds showed good biocompatibility, cell retention, and cell growth with spindle-like morphology five days post culture, mainly due to the enhanced intercellular signaling between cells. A cardiac patch must adhere well to the perpetual contracting and relaxing wall of the beating myocardium and must counter these stresses for better in vivo efficacy. To mimic this versatility, the chitosan/polyaniline conductive patch was stretched and contracted 1000 times and after two weeks the patch was quite adherent to the heart wall when implanted in vivo [56]. The proliferation of human and murine fibroblasts and murine C2C12 myoblasts increased significantly, when cultured on conductive Poly(l-lactide-co-ε-caprolactone)/PANI electrospun membranes [33]. H9c2 rat cardiomyoblasts cultured on conductive poly (l-lactide)/aniline pentamer films also demonstrated a high proliferation rate and, after six days under the electrical stimulation, cells demonstrated a 1.57-fold increase in the intracellular calcium concentration compared to the unstimulated group [51].

Pristine polyaniline [57], or when blended with non-conductive biomaterials [58,59], promoted the differentiation of the cultured stem cells to cardiomyocyte-like cells. An effect on cellular differentiation was observed in human mesenchymal stem cells and C2C12 cells cultured on PANI blended polycaprolactone (PCL) and silk fibroin conductive substrates. The electrical signals provided by the polyaniline needles stimulated the differentiation in mesenchymal stem cells as detected by the expression of sarcomeric α-actinin [36]. Interestingly, electrically conductive aligned PCL/PANI nanofibrous substrates guided the cellular orientation and differentiation of C2C12 myoblasts, promoting elongated myotube formation with high fusion and maturation indices compared to non-conductive substrates. This suggests the useful combined effect of the topographical and electrical cues for cell fate [44]. A more complex 3D cellular alignment and elongated myotube formation were observed when nanofiber yarn made of polycaprolactone, silk fibroin, and PANI was coated with a photocurable hydrogel. Long-term cultivation led to the striated morphology of the myotubes evident from the expression of structural myosin heavy chain (MHC) genes [47]. Another study demonstrated that H9c2 cells cultured on nanofibrous Poly(l-lactide acid)/PANI sheets generated myotubes with their length, myotube density, and fusion index increased 2–3 fold compared to when cultured on non-conductive PLA membranes. Furthermore, neonatal rat CMs cultured on these nanofibrous conductive PLA/PANI sheets maintained synchronous beating patterns for up to 21 days and exhibited striated morphology with enhanced expression of sarcomeric α-actinin and Cx43. Conversely, non-conductive PLA membranes induced irregular contractions in the cultured CMs [46].

Aligned conductive mesh of PANI and Poly(lactide-co-glycolic acid) attracted fibronectin and laminin from the culture medium and led to better cell attachment. Three days post culture, neonatal rat CMs developed rhythmic beating patterns with enhanced expression of cardiac troponin I (cTnI) and Cx43 [41]. Fibrous collagen-hyaluronic acid/PANI mats exhibited comparable electrical conductivity and superior mechanical properties to the myocardium. The conductive mats induced higher contraction amplitude and increased Cx43 expression with striated morphology in rat neonatal CMs [50]. Striated morphology was also observed when cells were cultured on Poly(glycerol sebacate)/aniline trimer conductive scaffolds. Conductive scaffolds significantly enhanced the expression of sarcomeric α-actinin and Cx43 compared to non-conductive PGS scaffolds. However, besides promoting synchronised and stronger beating patterns, the conductive scaffolds elicited a slight inflammatory response when subcutaneously implanted in rats for thirty days [49]. In another study, copolymer produced by hyperbranched Poly(l-lactide) blended with aniline tetramer demonstrated good biodegradability, high ductility and high electroactivity. After seven days of culture, these conductive copolymers promoted the myogenic differentiation of C2C12 cells to myotubes with increased myotube density, length, diameter, and maturation index also evident from the elevated expression of MyoD and cTnT2 [54].

More recently, conductive injectable hydrogels based on dextran grafted aniline tetramer blended with chitosan demonstrated adequate in vivo injectability and degradability when implanted subcutaneously in rats. Different cell lines, such as C2C12 and L929, encapsulated in these conductive hydrogels maintained vitality and proliferation capacities when released in a rat volumetric muscle loss injury model. Therefore, these conductive hydrogels could also be used as a cell delivery vehicle, promoting muscle tissue regeneration [55].

### 2.2. Polypyrrole

Polypyrrole (PPy) is another intrinsically conductive polymer that has been extensively studied in the field of biomedical and tissue engineering, as shown in Table 2 [60,61,62,63,64,65]. It has been reported that polypyrrole offers more favourable biological properties than polyaniline. However, under similar experimental conditions, cells cultured on these two polymeric substrates have demonstrated comparable biological responses [66]. PPy can be chemically modified and combined with other polymers to provide a biocompatible electrical microenvironment to the cells. This way, cell viability, proliferation rate, and intercellular coupling can be increased appreciably with a high potential to direct their differentiation toward cardiomyocyte-like phenotype. Poly(L-lactide-co-glycolic acid)/PPy conductive 3D electrospun scaffolds exhibited good biocompatibility when cultured with mouse cardiac progenitor cells and human-induced pluripotent stem cells (hiPSCs). Induced pluripotent stem cells proliferated along the conductive fibre lengths without severe apoptosis up to ten days post-culture [67]. Likewise, PPy-chitosan hydrogels offered no cytotoxicity to rat smooth muscle cells and promoted their proliferation, morphology, and metabolism. These conductive hydrogels significantly enhanced the intracellular Ca^2+^ transient propagation in cultured neonatal rat CMs and improved myocardial electrical signal conduction with elevated cardiac functions in the rat MI model [68]. Human-induced pluripotent stem cells derived CMs demonstrated a sarcomeric length of 1.86 µm three weeks post culture on nanopatterned silk fibroin/PPy conductive scaffolds [69] while the average sarcomeric length of human CMs in a relaxed state is about 2.2 µm [70]. This demonstrates the ability of the nanopatterned conductive scaffold to achieve near-physiological sarcomeric lengths owing to nanoscale channels which directed the unidirectional growth and proliferation of cells to achieve cardiac muscle-like striated morphology. Seven days post culture, hiPSCs differentiated to cardiomyocyte-like cells with Nkx2.5 and GATA4 expression elevated by 2 and 3.3-fold, respectively, when cultured on Poly(lactide-co-glycolic acid)/PPy conductive membranes [18].

Calcium is a critical regulator of the cardiac contraction and relaxation cycle. Intracellular Ca^2+^ influx called calcium transient is the link between the electrical signals that run throughout the heart and myocyte contractions to pump blood [75]. HL-1 murine CMs cultured on conductive PCL/PPy films improved the propagation of calcium transients with high conduction velocity twelve days post culture. Transient recovery time (time to recover 50% intracellular Ca^2+^ levels) was reduced by 1.24-fold compared to cells cultured on non-conductive PCL films [71,76]. Similarly, hiPSCs grown on conductive silk fibroin/PPy scaffolds differentiated to cardiomyocyte-like cells and significantly upregulated their action potentials after three weeks [69]. In addition, cells demonstrated an enhanced expression of Cx43, Myh7, cTnT2, and SCN5A genes compared to when cultured on non-conductive silk fibroin membranes. Myh7 is one of the myosin heavy chain genes that supports cell contraction [77]. The action potentials fired by CMs propagated easily due to the conductive nature of the scaffold and resulted in ionic to electronic current transition. This electronic current tends to open the voltage-gated sodium channels Na_v_1.5 encoded by the SCN5A gene [78]. The influx of Na^+^ ions then depolarised the plasma membrane, leading to the synchronous beating of the CMs.

### 2.3. Poly(3,4-Ethylenedioxythiophene)/PEDOT

PEDOT-based electroconductive scaffolds represent a good electrical interface with biological cells/tissues owing to their intrinsic capability to conduct electrical currents, good stability, mechanical properties and biocompatibility, thus providing promising platforms for tissue engineering applications [79,80,81]. To enhance the poor water solubility, it is combined with Poly(styrenesulfonic acid)/PSS in a polymer blend. A conductive PEDOT:PSS/PEG hydrogel was prepared using two-step sequential polymerisation by in situ synthesis of PEDOT within the pre-crosslinked PEG hydrogel. To promote cell adhesion, RGD peptides were covalently attached to the conductive hydrogel surface. In vitro studies with H9C2 myocytes demonstrated good biocompatibility of this conductive hydrogel. It also supported cell adhesion and proliferation up to five days of culture [82]. Better results to promote cardiomyogenic differentiation have been obtained culturing cardiac cells on more complex conductive structures mimicking the fibrous and porous nature of cardiac ECM. A material mimicking elastomeric mechanical properties of the cardiac ECM was obtained by interpenetrating PEDOT into nitrile butadiene rubber (NBR) and Poly(ethylene glycol) dimethacrylate (PEGDM) crosslinked electrospun mats. PEDOT-embedded mats displayed high flexibility and conductivity, and induced enhanced expression of Cx43 and α-actinin in cardiomyocytes after five days of incubation, suggesting the presence of contractility and cell maturation [83].

A highly porous PEDOT/alginate conductive scaffold was prepared to form a chemically crosslinked alginate network, using adipic acid hydrazide as the crosslinker, in which simultaneously PEDOT was synthesized in situ. Brown adipose-derived stem cells (BADSCs) differentiated to cardiomyocyte-like cells seven days post-culturing on these conductive porous scaffolds. Almost 21% and 60% of the adherent cells demonstrated enhanced expression of Cx43 and cTnT2, respectively, with most cells exhibiting enhanced α-actinin expression. The electrical stimulation improved the differentiation of BADSC to cardiomyocytes on these porous scaffolds [84]. Likewise, conductive constructs with a fibrous microstructure prepared as PEDOT:PSS/collagen-alginate hydrogels directed the myocardial differentiation of hiPSCs to cardiomyocytes-like cells. Eleven days post culture, cells demonstrated enhanced expression of cTnI and α-actinin. Cells also started to beat with the contraction amplitude increased by 1.9-fold and average sarcomeric length by 1.26-fold compared to when cells were cultured on non-conductive collagen-alginate hydrogels [85].

To further mimic the micro/nanofibrous architecture of the native myocardium as well as the myocardial electrical conductivity, a multiscale conductive scaffold was fabricated via a layer-by-layer deposition approach combining solution-based and melt-based electrohydrodynamic (EHD) printing techniques. PCL microfibers were printed to mimic collagenous fibres and sub-micron scale conductive PEDOT:PSS-PEO fibres were printed to mimic the electrically conductive Purkinje fibres of native cardiac tissue. Fibre orientation was controlled in a layer-by-layer manner. Primary rat cardiomyocytes demonstrated good adhesion with PEDOT:PSS-PEO/PCL micro/nanofibrous conductive scaffolds and, after eight days, cells exhibited enhanced expression of α-actinin and Cx43. Cells started to beat synchronously and the beating frequency increased by 1.46-fold compared to cells cultured on non-conductive PCL scaffolds [86]. Similarly, cardiomyocytes displayed excellent adhesion and proliferation rate when cultured on conductive PEDOT/alginate-collagen hydrogels. Eleven days post culture, cells started to beat synchronously with a 10.5-fold increase in the beating frequency with the expression of cTnI and Cx43 twice as high as when cells were cultivated on non-conductive hydrogels. The non-conductive hydrogels induced arrythmias in the cells [85]. Table 3 summarizes the applications of PEDOT-based conductive constructs in cardiac tissue engineering and their effect on cellular response.

## 3. The Underlying Mechanisms of the Positive Role of Conductive Substrates in Cardiac Tissue Engineering

**Figure 3:**
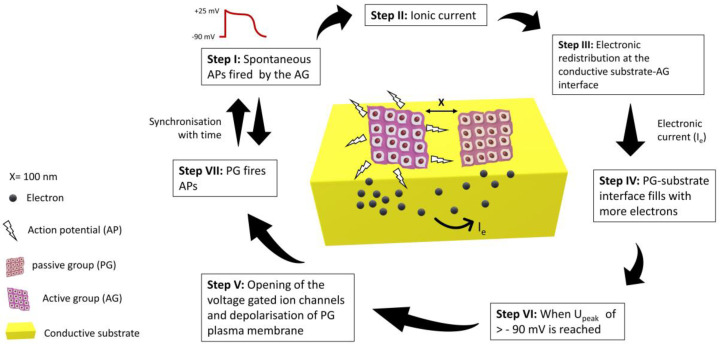
A theoretical model of ionic to electronic current transition for cells cultured on a conductive substrate. The ionic current interacting with electrons could direct their flow in other directions. This electronic current, opening the voltage-gated ion channels, would result in the influx of Na^+^, K^+^ and Ca^2+^ ions. This influx will depolarise the plasma membrane and the passive group (PG) will eventually fire action potentials.

Though the electroconductive scaffolds provide better platforms for the tissue engineering of nerve, cardiac, and skeletal muscle tissues, the mechanism by which these constructs influence cellular behaviour is yet to be known [88]. To uncover this, an equivalent circuit model was proposed for two groups of cardiomyocytes seeded on a conductive substrate. One group was assumed to be active (AG), which could fire spontaneous action potentials, while the other was assumed to be passive (PG), which could not fire spontaneous action potentials, but it was well electrically coupled. AG was considered the only source of ionic current, and the passive group was only affected by the electrical currents from the neighbouring AG. Nano gaps/clefts are formed between the cell membrane of the cultured cardiomyocytes and the substrate surface. Ionic solution filled these clefts during culturing, and it was modelled as seal resistance [89]. Seal resistance is usually generated by the solution between cell-substrate interfacial gaps and has a direct link with the adhesion strength of cells with the substrate [90]. When cells in the active group (AG) fire spontaneous action potentials, the electronic redistribution on the surface of the conductive substrate takes place just beneath the cells. The interfacial clefts between PG cells and substrate fill with more anions due to this electronic redistribution, which depolarises the plasma membrane of cells and could trigger an action potential. Larger seal resistance gives rise to higher excitation potential (U_peak_) which indicates a strong electrical coupling between AG and PG. The passive group will fire an action potential once the threshold (U_peak_) crosses the resting membrane potential of −90 mV [91,92]. In this way, PG could be stimulated and electrically synchronised to AG under the influence of action potentials generated by the active group, as shown in Figure 3. Recent studies have confirmed that the conductive scaffold can decrease the resistivity of the fibrotic scar tissue in the infarcted region [25,93,94,95]. In one of the studies, the Langendorff-perfused beating heart as the source of ionic current was placed on one side and a microelectrode array (MEA) was placed on the other side to measure the field potential amplitude, with a gelatin matrix cushion in the middle mimicking the inert scar tissue. When there was only gelatin between the beating heart and MEA, the field potential was hardly detected (125 µV). On the other hand, the field potential increased by 10-fold when the gelatin cushion was filled with conductive chitosan/polypyrrole conductive solution. This suggests that the ionic current (action potentials) generated by the beating heart was able to cause electronic re-distribution and triggered an electric current, which travelled through the conductive solution and was ultimately detected by MEA. This conductive solution was able to lower the resistivity of the scar tissue by 1.63-fold compared to when induced MI rat hearts were injected with non-conductive chitosan. Twelve weeks post-injection, the field potential was twice as high as when the hearts were injected with pure chitosan. The electric pulse travelled throughout the scar tissue with 60 cm/s of conduction velocity, while in chitosan injected hearts it was partially blocked by non-conductive scar tissue and could not travel through the entire tissue [25].

## 4. Conductive Substrates for In Vivo Cardiac Repair

Electroconductive scaffolds represent a leap forward in the effort to manufacture supports to properly address cell fate towards a cardiomyocytic phenotype. The scaffold fabrication is a complex endeavour finalised to replicate in vitro the ECM microenvironment to foster proper cell-cell and cell-matrix interactions driving the cardiomyocyte maturation process. An optimal scaffold must be biocompatible (but not necessarily biodegradable, if the constituent materials are immunopermissive), with (i) adequate porosity (to deliver biologically active factors and remove cell waste) and (ii) mechano-structural design (to deliver physical signals, such as stiffness, micro/nano-topography, etc.), and (iii) with electroconductivity appropriate to mimic the ECM structure and function. Finally, these characteristics must allow the integration of the engineered tissue with the native injured myocardium to restore heart function. Studies on experimental animals have shown that scaffolds endowed with appropriate electroconductivity substantially improve the beneficial effects after implantation in injured hearts. Ejection fraction (EF) and fraction shortening (FS) that give a measure of the blood pumping ability of the heart [96,97] were improved after three months when induced-MI rat hearts were injected with conductive chitosan/PPy hydrogels [25]. Infarcted tissue demonstrated improved angiogenesis when injected with conductive alginate/PPy hydrogels. The number of myofibroblasts infiltrating the infarcted area was 1.4 times greater than when hearts were injected with non-conductive alginate hydrogels [98]. Contractile myofibroblasts expressing α-SMA (smooth muscle actin) can restore the structural integrity of the infarcted myocardium and could also help in restoring the cardiac functions by reducing the scar size [99,100,101,102,103]. Similarly, blood vessel density increased by 2.6-fold when a conductive chitosan/PPy patch was implanted on the infarcted wall [72]. A reduction in scar size and the regeneration of the injured ventricular wall are the deciding factors for the in vivo efficacy of a conductive substrate. When injected with gelatin-hyperbranched poly(amino ester)/PPy hydrogels, the infarct size was reduced by 3.4-fold while LV wall thickness increased by 2.6-fold four weeks post-injection [74]. A new class of emerging conductive polymeric hydrogels made of poly-3-amino-4-methoxybenzoic acid/gelatin (PAMB-G) increased the electrical pulse propagation through scar tissue with a 1.6-fold increase in the conduction velocity [94]. When loaded with CMs, the PAMB-G patch reduced the scar resistivity evident from a 3.4-fold increase in the field potential. The electric pulse propagated twice as fast as compared to when MI heart was injected with gelatin alone. A cell-laden conductive patch also led to improved tissue regeneration with reduced scar size and shorter QRS intervals [95]. Table 4 shows the in vivo cardiac repair efficacy of the conductive scaffolds.

These encouraging results, however, do not clarify the mechanisms through which the engineered tissue is integrated into the injured myocardium. After an ischemic attack, the deprivation of the oxygen and other blood-borne factors determine the impairment of the myocardial machinery (tissue pH and intra-cellular cascades are undermined, and necrotic and apoptotic processes are activated) subverting the ECM composition and function. Among others, a complex array of inflammatory factors is released that induces the recruitment of different immune cell populations. Macrophages are accumulated in the damaged tissue either to remove the debris (M1 population) or to activate the limited native regenerative potential (M2 population). As a final result, myocardial bioarchitecture is wrecked, the vascular beds are no more hierarchically juxtaposed, and fibrosis alters the tissue stiffness and other mechano-structural characteristics. In this inhospitable context, it is hard to imagine that the grafted cells/structure viability can be adequate to be integrated with the host tissue. This requires that novel strategies are implemented to modulate the processes occurring in the injured myocardial tissue. The electroconductive scaffold can be used as a device to release novel drugs and to apply mechano-structural and electric signals able to restrain the overwhelming inflammatory processes. At the same time, an electroconductive scaffold can enhance the cardiomyocytic differentiation potential of the MSC, hiPSCs, and CPC (cardiac progenitor cells) used to manufacture the engineered tissue. In fact, MSCs do not express the MHC class II molecules and thus evade immune system recognition. They also depress the inflammatory reaction modifying the polarisation of M1 macrophages to reparative M2 macrophages via miR-182 shuttling [105] and decreasing neutrophil infiltration and T-cell proliferation [106]. The electroconductive scaffold can provide the “cables” through which a more physiologic treatment can be applied to the post-ischemic myocardium.

## 5. Concluding Remarks

Hopes that the adult human heart can regenerate after infarction remain scarce due to the low turnover rate of the cardiomyocytes. Therefore, cardiac tissue engineering could provide promising solutions to repair the injured myocardium. Culturing stem/progenitor cells on electroconductive scaffolds can increase their differentiation by potentially several folds due to the electrical properties of such scaffolds. As the intrinsic electric field of the conductive scaffold encourages the differentiation of stem cells to cardiomyocyte-like cells, this eventually leads to the expression of the key cardiac proteins, such as Cx43, and subsequently facilitates the electrical coupling and contractility of cells. Cardiac functions of the infarcted heart are significantly alleviated as well when the conductive scaffold is implanted in vivo, as shown in rat MI models (Table 4). This significant contribution of conductive polymer-based cardiac tissue engineering to repair myocardial infarction is alluring, but some challenges remain. Even though the anti-bacterial [48] and antioxidant properties [40] of conductive polymers may seem attractive features for tissue engineering applications, their non-biodegradability [107,108,109] and potential to elicit cytotoxicity if used in high concentrations [37,43,73,87,110] make them unsafe for long term use. The non-biodegradable debris could elicit the inflammatory response at the local tissue site resulting in immune rejection. Therefore, a biodegradable, intrinsically conductive polymer whose conductivity could be tuned to mimic the electrical features of the myocardium is of great necessity in cardiac tissue engineering. No material has ever been produced which would undergo perpetual contraction and relaxation cycles for an entire span of natural life, like myocardium. To mimic this versatility, future studies should take into consideration the complex biomechanics, anisotropy, micro/nano-architecture, and electrical properties of the myocardium.

## Figures and Tables

**Table 1:** Polyaniline based conductive constructs for cardiac tissue engineering.

Conductive Substrate	Mechanical Properties	Electrical Properties	Cell Line or Tissue	Biological Response
Poly-l-Lysine-PANI nanotubes membranes [32]			Rat CMs	Better CMs proliferation
PLCL, PANI electrospun membranes [33]	E = 50 MPa, ε_r_ = 207.85%, UTS = 0.69 MPa	Four-probe technique, σ = 13.8 mS/cm	Human fibroblasts, NIH-3T3, C2C12	Improved cell adhesion and metabolic activity
PGLD, PANI nanotubes membranes [34]			Cho cells, neonatal rat CMs	Good biocompatibility
PU-AP/PCL porous scaffold [35]	E_c_ = 4.1 MPa, C.S = 1.3 MPa	Four-probe technique, σ = 10^−5^ S/cm	Neonatal rat CMs	Enhanced Actn4, Cx43, and cTnT2 expressions.
PANI/PCL patch [36]		Two-probe technique, σ = 80 µS/cm	hMSCs	Differentiation of hMSCs to CM-like cells
PDLA/PANI electrospun membranes [37]		σ = 44 mS/cm	primary rat muscle cells	Improved cell adhesion and proliferation
Gelatin/PANI electrospun membranes [38]	E = 1384 MPa, UTS = 10.49 MPa, ε_r_ = 9%	Four-probe technique, σ = 17 mS/cm	H9c2	Smooth muscle-like morphology rich in microfilaments
Gelatin/PANI hydrogels [39]	G’ = 5 Pa, G” = 26 Pa	Pocket conductivity meter, σ = 0.45 mS/cm	C2C12, BM-MSCs	Improved cell-cell signalling and proliferation
PU-AP/PCL films [40]	E’ = 10 MPa at 37 °C	Four-probe technique, σ = 10^−5^ S/cm	L929, HUVECs	Improved cytocompatibility, good antioxidant properties
PLGA, PANI electrospun meshes [41]	E = 91.7 MPa	Four-point probe, σ = 3.1 mS/cm	Neonatal rat CMs	Enhanced Cx43 and cTnI expressions
PGS/PANI composites [42]	E = 6 MPa, UTS = 9.2 MPa, ε_r_ = 40%	Four-probe technique, σ = 18 mS/cm	C2C12	Good cell retention, growth, and proliferation
PCL, amino capped AT films [43]	E = 31.2 MPa, UTS = 48.3 MPa, ε_r_ = 646%	-	C2C12	Spindle like morphology, myotube formation
PCL, PANI electrospun membranes [44]	E = 55.2 MPa, UTS = 10.5 MPa, ε_r_ = 38.0%	Four-point probe, σ = 63.6 mS/cm	C2C12	Myotube formation
PANI, E-PANI films [45]		Z > 10 MΩ/sqr for PANI Z = 6 MΩ/sqr for E-PANI	H9c2	Improved proliferation and cell attachment on E-PANI
PLA/PANI electrospun membranes [46]		Four-probe technique, σ = 21 µS/m	H9c2, rat CMs	Myotube formation from H9c2 cells, enhanced Cx43 and α-actinin expression, improved Ca^2+^ transients for CMs
PCL/SF/PANI hydrogels [47]	ε_r_ = 107%		C2C12	Excellent cell alignment, myotube formation
Chitosan-AT/PEG-DA hydrogels [48]	G’ = 7 kPa	Pocket conductivity meter, σ = 2.42 mS/cm	C2C12, H9c2	Improved cell viability
PGS-AT elastomers [49]	E = 2.2 MPa, UTS = 2.0 MPa, ε_r_ = 141%	-	H9c2, rat CMs	Synchronous CM beating with improved Ca^2+^ transients, H9c2 showed good orientation, enhanced Cx43 and α-actinin expression
PANI, Collagen, HA electrospun mats [50]	E = 0.02 MPa, UTS= 4 MPa, ε_r_ = 78%	Four-probe technique, σ = 2 mS/cm	Neonatal rat CMs, hiPSCs	Synchronous beating of CMs derived from hiPSCs. Enhanced Cx43 and cTnI expression
AP, PLA films [51]		Four-point probe, σ = 10^−6^ to 10^−5^ S/cm	H9c2	Pseudopodia like morphology, improved Ca^2+^ transients
Chitosan, PANI patch [52]	E = 6.73 MPa, UTS = 5.26 MPa, ε_r_ = 79%	Four-probe technique, σ = 0.162 S/cm	Rat MI heart	Improved CV in the infarcted region with healing effects
PA, PANI patch [53]	Elongation = 84%	Digital Avometer, σ = 2.79 S/m	Pork heart	Cardiac ECM mimicking
HPLA/AT films [54]	ε_r_ = 42.7%, E = 758 MPa		C2C12	Myotube formation
Dextran-AT/chitosan [55]	G’ = 620 Pa at t = 50 min	Four-probe technique, σ = 0.03 mS/cm	L929, C2C12	high proliferation rate, good in vivo degradation, generation of new myofibers

Abbreviations: PANI: Polyaniline, PLCL: poly(l-lactide-*co*-ε-caprolactone), E: elastic modulus, ε_r_: strain at rupture, UTS: ultimate tensile strength, E_c_: compressive modulus, C.S: compressive strength, G’: shear storage modulus, G”: shear loss modulus, E’: tensile storage modulus σ: electrical conductivity, CMs: cardiomyocytes, PU: polyurethane, AP: aniline pentamer, PCL: polycaprolactone, hMSCs: human mesenchymal stem cells, hiPSCs: human induced pluripotent stem cells, PDLA: poly (d-lactic acid), BM-MSCs: bone marrow-derived mesenchymal stem cells, PGS: polyglycerol sebacate, AT: aniline tetramer, E-PANI: polyaniline-emeraldine base, PLA: polylactic acid, SF: silk fibroin, PEG-DA: dibenzaldehyde-terminated poly(ethylene glycol), HA: hyaluronic acid, PA: polyamide, HPLA: hyperbranched polylactide, CV: conduction velocity

**Table 2:** Polypyrrole based conductive constructs in cardiac tissue engineering.

Conductive Substrate	Mechanical Properties	Electrical Properties	Cell Line or Tissue	Biological Response
PCL, PPy films [71]	Nanoindentation test, E = 0.93 GPa	Keithley Parameter Analyzer, ρ = 1.0 kΩ-cm	HL-1 murine CMs	Enhanced Cx43 expression, improved Ca^2+^ transients
Chitosan, PPy porous membranes [72]	E = 486.7 kPa	Three-probe detector, σ = 63 mS/m	NRVMs, rat MI model	Improved cytoskeletal organisation with high beating amplitude, tissue morphogenesis at the MI site
SF, PPy composites [69]	E = 200 MPa, UTS = 7 MPa	Four-probe technique, σ = 1 S/cm	hPSC-CMs	Enhanced expression of Cx43, Myh7, cTnT2, SCN5A genes, elongated Z-band width and sarcomeric length
Chitosan, PPy hydrogels [68]	E = 3 kPa	Four-point probe, σ = 0.23 mS/cm	Neonatal rat CMs, rat SMCs	Good proliferation with elevated calcium transients and shorter QRS intervals
PLGA, PPy membranes [67]			Mice CPCs, hiPSCs	Good biocompatibility and proliferation rate
PLGA, PPy membranes [18]			hiPSCs	Differentiation of hiPSCs to CMs, enhanced expression of actinin, Nkx2.5, GATA4, and Oct4
PCL, gelatin, PPy electrospun membranes [73]	E = 50.3 MPa, ε_r_ = 3.7%	Four-probe technique, σ = 0.37 mS/cm	Rabbit primary CMs	High proliferation rate, enhanced expression of Cx43, cTnT, and α-actinin
PPy, HPAE hydrogels [74]	G’ = 35 kPa,	Four-probe technique, σ = 0.65 mS/cm	L929, BMSCs	Enhanced Cx43, α-SMA expressions, excellent cell viability and biocompatibility

Abbreviations: PPy: polypyrrole, E: elastic modulus, ε_r_: strain at rupture, G’: shear storage modulus, σ: electrical conductivity, NRVMs: neonatal rat ventricular myocytes, hPSCs-CMs: human pluripotent stem cells derived cardiomyocytes, SF: silk fibroin, CPCs: cardiac progenitor cells, hiPSCs: human induced pluripotent stem cells, PLGA: poly(lactic-co-glycolic acid), HPAE: hyperbranched poly(amino ester), BMSCs: bone marrow-derived mesenchymal stem cells, α-SMA: alpha-smooth muscle actin.

**Table 3:** PEDOT based conductive constructs in cardiac tissue engineering.

Conductive Substrate	Mechanical Properties	Electrical Properties	Cell Line	Biological Response
PEG/PEDOT:PSS hydrogels [82]	E_c_ = 21 kPa	Four-probe technique, σ = 16.9 mS/cm	H9c2	Good cell viability and proliferation
GelMA/PEDOT:PSS hydrogels [87]	E = 10.3 kPa	ElS, Z = 261 kΩ at 1 Hz	C2C12	Good cell viability and proliferation but high polymer concentration was detrimental to cells
Collagen/alginate/ PEDOT:PSS hydrogels [85]	G = 220 Pa, τ_max_ = 41 Pa	Four-probe technique, σ = 3.5 mS/cm	CMs, hiPSCs-CMs	Good cell viability, proliferation, and adhesion, synchronous beating patterns
Alginate/PEDOT hydrogels [84]	E_c_ = 175 kPa, G’ = 100 kPa, G” = 10 kPa	Electrochemical workstation, σ = 61 mS/cm	BADSCs	Differentiation of BADSCs to CMs with enhanced expression of cTnT, α-actinin, Cx43
NBR/PEGDM/PEDOT electrospun membranes [83]	E = 3.8 MPa, ε_r_ = 75.1%	Four-probe technique, σ = 5.8 S/cm	Cardiac fibroblasts	Well organised sarcomeres, enhanced expression of α-actinin, Cx43
PCL/PEDOT:PSS microfibrous scaffold [86]	E = 13 MPa		H9c2, primary CMs	Enhanced expression of Cx43 and α-actinin, synchronous beating patterns of CMs

Abbreviations: E: elastic modulus, E_c_: compressive modulus, ε_r_: strain at rupture, G: shear modulus, G’: shear storage modulus, G”: shear loss modulus, τ_max_: maximum shear stress, σ: electrical conductivity, PEG: polyethylene glycol, EIS: electrochemical impedance spectroscopy, BADSCs: brown adipose-derived stem cells, NBR: nitrile butadiene rubber, PEGDM: poly(ethylene glycol) dimethacrylate, GelMA: gelatin methacrylate

**Table 4:** Preclinical studies using rat MI models to repair the ischemic tissue.

Conductive Substrate	Outcomes
Chitosan, PANI patch [52]	CV increased from 24.3 cm/s in the apex of the MI zone to 30.1 cm/s after applying the conductive patch.
Chitosan, PANI auxetic patch [104]	Minimal in vivo fibrotic response, good adhesion with the heart wall, no significant improvements in the cardiac functions though.
Alginate, PPy hydrogel [98]	**Conductive hydrogel injected hearts:** Increased angiogenesis with arteriole density of 33 arteriole/µm^2^, five weeks post-injection. **PBS injected hearts:** low arteriole density of 21 arteriole/µm^2^.
Chitosan, PPy hydrogel [25]	**Conductive hydrogel injected hearts:** EF/FS improved to 67%/34%, QRS/QT intervals reduced to 13/42 ms, and high CV of 62 cm/s, three months post-injection. **Saline injected hearts:** EF/FS was around 53%/25%, prolonged QRS/QT intervals of 17/70 ms, CV dropped to 43 cm/s.
Chitosan, PPy hydrogel [68]	Improved electrical signal conduction in the infarcted zone with reduced QRS intervals.
Chitosan, PPy patch [72]	**Patch implanted hearts:** Enhanced angiogenesis with 180 blood vessels/mm^2^, tissue regeneration area of 0.3 µm^2^, four weeks post-implantation. **Chitosan injected hearts:** Reduced angiogenesis with 68 blood vessels/mm^2^ and tissue regeneration area of 0.2 µm^2^.
PPy, HPAE, gelatin hydrogel [74]	**Conductive hydrogel injected hearts:** EF/FS improved to 56%/31%, EDV/ESV to 325/150 µL, QRS interval reduced to 16 ms, after four weeks of injection. **MI hearts:** EF/FS dropped to 29%/15%, EDV/ESV to 525/420 µL, prolonged QRS of 40 ms.
PPy, chitosan hydrogel [93]	**Conductive hydrogel injected hearts:** Improved longitudinal CV of 74.3 cm/s through the scar tissue after seven days of injection, shorter QRS interval of 18.6 ms, four weeks post-injection. **Saline injected hearts:** longitudinal CV dropped to 57.7 cm/s, prolonged QRS interval of 23.7 ms.
PAMB-G hydrogel [94]	**Conductive hydrogel injected hearts:** Regional field potential increased to 1.5 mV and CV to 40 cm/s in the scar tissue, EF/EF improved to 67%/31%, LVIDs and LVIDd reduced to 6.2 mm and 8.7 mm, respectively, four weeks post-injection. **Gelatin injected hearts:** Regional field potential was around 0.7 mV with a slow CV of 27 cm/s, EF/FS dropped to 56%/23%, LVIDs and LVIDd were around 7 mm and 9.3 mm, respectively.
PAMB-G patch [95]	**Patch implanted hearts:** Regional field potential increased to 1.9 mV with a CV of 39 cm/s in the scar tissue, reduced QRS intervals of 13 ms, EF/FS improved to 60%/28%, LVIDs and LVIDd reduced to 5.3 mm and 7.8 mm, respectively, four weeks post-implantation. **Gelatin injected hearts:** Reduced regional field potential of 0.7 mV with a slow CV of 19 cm/s, prolonged QRS intervals of 22 ms, EF/FS dropped to 57%/26%, LVIDs and LVIDs were around 5.6 mm and 8.2 mm.

Abbreviations: EDV: end-diastolic volume, ESV: end-systolic volume, ESA: end-systolic area, EDA: end-diastolic area, LVEDD: left ventricular end-diastolic diameter, LVESD: left ventricular end-systolic diameter, LVIDs: left ventricular internal diameter at end-systole, LVIDd: left ventricular internal diameter at end-diastole, EF/FS: ejection fraction/fraction shortening, PEG-MEL: polyethylene glycol-melamine, PAMB-G: Poly-3-amino-4-methoxybenzoyic acid grafted on gelatin, HPAE: hyperbranched poly(amino ester).
